# The Combination of Ketorolac with Local Anesthesia for Pain Control in Day Care Retinal Detachment Surgery: A Randomized Controlled Trial

**DOI:** 10.1155/2017/3464693

**Published:** 2017-07-09

**Authors:** Xiaohong Chen, Bingqian Liu, Xiaoling Liang, Jiaqing Li, Tao Li, Yonghao Li, Xiling Yu, Cancan Lyu, Xiujuan Zhao, Silvia Tanumiharjo, Chenjin Jin, Lin Lu

**Affiliations:** State Key Laboratory of Ophthalmology, Zhongshan Ophthalmic Center, Sun Yat-sen University, Guangzhou, China

## Abstract

This study aims to evaluate the efficacy of ketorolac with local anesthesia compared to local anesthesia alone for perioperative pain control in day care retinal detachment surgery. The randomized controlled trial included 59 eyes of 59 participants for retinal detachment surgery who were randomly assigned (1 : 1) into the ketorolac (K) group and control (C) group. All participants underwent conventional local anesthesia while patients in the K group received an extra administration of preoperative ketorolac. Participants in the K group had a statistically significantly lower intraoperative NRS score (median 1.0 versus 3.0, *P* = 0.003), lower postoperative NRS score (median 0 versus 1.0, *P* = 0.035), fewer proportion of rescue analgesic requirement (10% versus 34.5%, *P* = 0.023), and lower incidence of postoperative nausea and vomiting (13.3% versus 41.4%, *P* = 0.015) compared to the C group. Intraocular pressure (IOP) changes (△IOP) were significantly reduced in the K group (median 1.9 versus 3.0, *P* = 0.038) compared to the C group 24 hours postoperatively. In conclusion, the combination of local anesthesia with ketorolac provides better pain control in retinal detachment surgery compared to local anesthesia alone. The beneficial effect of ketorolac with local anesthesia may contribute to a wider-spread adoption of day care retinal detachment surgery. This trial is registered with ClinicalTrials.gov NCT02729285.

## 1. Introduction

Rhegmatogenous retinal detachment (RRD) with a reduction of visual acuity (VA) is an indication for surgical treatment. Although pars plana vitrectomy (PPV) has gained a high popularity and is regarded as the best approach for RRD by the majority of ophthalmologists, scleral buckling (SB) surgery has been proved as effective as PPV in uncomplicated RRD in a meta-analysis of prospective randomized trials by Soni et al. [1]. The authors reported that the postoperative BCVA was better in the eyes treated by SB than those treated by PPV, probably because of a higher rate of cataract formation in the eyes of PPV treatment. Therefore, more attention needs to be paid to SB in the uncomplicated RRD patients [2, 3], especially in those RRD patients without posterior vitreous detachment (PVD) [4].

The practice of local anesthesia (LA) to SB surgery has been increased in recent years [5, 6], especially in the setting of a day care unit. LA is more beneficial to the performance of operations in a day care unit for lower medical cost, lower risks of anesthetic accident, and quicker recovery compared to general anesthesia (GA) [7]. Nevertheless, several reasons may contribute to the perioperative discomfort despite an administration of LA: tissue division [8], repeated ocular muscular traction [9], cryopexy [10], and inflammation [8, 9]. In order to provide a better anesthetic and analgesic environment for surgery, we chose ketorolac as an adjuvant to local anesthesia in day care retinal detachment surgery.

Ketorolac belongs to the family of nonsteroidal anti-inflammatory drugs (NSAIDs) and has been confirmed a short-term analgesic as effective as morphine [11, 12]. Grimsby et al. [11] reported that the continuous infusion of ketorolac offered a good pain control after renal surgery. Besides, Yadav et al. [13] reported a significantly improved anesthetic efficacy via preoperative ketorolac with buccal and lingual infiltration combined with articaine inferior alveolar nerve block in mandibular molars with irreversible pulpitis. Moreover, Kim et al. [14] showed that preoperative ketorolac could effectively reduce postoperative pain in laser-assisted subepithelial keratectomy (LASEK). In light of the concept of pain-free anesthesia during the surgery, we designed this trial to evaluate the efficacy of ketorolac with LA compared to LA alone for patients' pain relief in day care retinal detachment surgery.

## 2. Patients and Methods

This trial was conducted at the Zhongshan Ophthalmic Center, Sun Yat-sen University, Guangzhou, China. Ethical approval for this study (identifier: 2014MEKY042) was obtained by the Ethics Committee of Zhongshan Ophthalmic Center, Sun Yat-sen University, China, and informed consent was obtained from each enrolled subject. The trial was registered with ClinicalTrial.gov NCT02729285 and adhered to the tenets of the Declaration of Helsinki.

### 2.1. Participants

**Figure 1:**
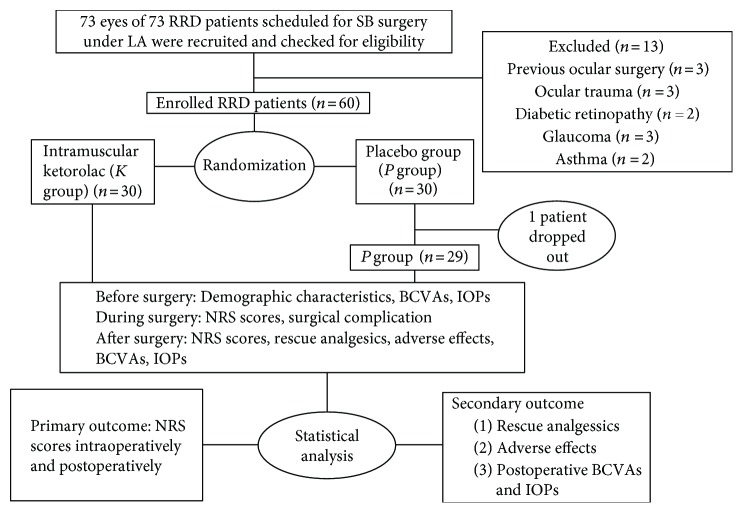
A flow chart showing the enrollment, assignment, procedures, outcome assessments, and data analysis during the whole study. RRD = rhegmatogenous retinal detachment; SB = scleral buckling; LA = local anesthesia; BCVA = best-corrected visual acuity; IOP = intraocular pressure; NRS = Numerical Rating Scales.

Our study recruited a total of 73 eyes of 73 adult participants diagnosed with RRD that were scheduled for scleral buckling surgery under a retrobulbar block of LA in a day care unit. The participants included were those 18 years old or above, with a body mass index (BMI) of 18.5–24 kg/m^2^ [15], American Society of Anesthesiologists (ASA) physical status I or II, and an understanding of the 11-point Numerical Rating Scales (NRS) [16]. The participants were excluded as follows: history of ocular surgery, trauma, or infection; glaucoma or diabetic retinopathy; diagnosis of renal or liver impairment; diagnosis of asthma, allergy, or coagulopathy; chronic pain syndromes; history of peptic ulceration; history of chronic use of analgesics, sedatives, opioids, or steroids; history of drug or alcohol abuse; history of sexually transmitted disease (STD), including hepatitis B diseases, tuberculosis, syphilis, and acquired immune deficiency syndrome(AIDS); pregnancy or lactation; and cognitive impairment or psychiatric illness. A flow chart is presented for the whole study procedures (Figure 1).

### 2.2. Randomization and Masking

By using computer-generated randomization, the participants enrolled were allocated (1 : 1) to the ketorolac group (K group) and control group (C group). Both groups received the administration of intramuscular hemocoagulase 30 minutes before surgery for preoperative preparation. Patients in the K group received an intramuscular injection of ketorolac (Lunan Pharmaceutical Group Corporation, Linyi, Shandong Province, China) 30 minutes before surgery. The patients and the outcome evaluators were blind to the randomization.

### 2.3. Demographic Characteristics and Ophthalmic Examinations

The participants' demographic characteristics including ages and genders were documented. Each participant enrolled in our trial had a comprehensive ophthalmic examination including best-corrected visual acuity (BCVA), intraocular pressure (IOP), and a slit-lamp evaluation preoperatively and 24 hours postoperatively. The BCVA was converted into the logarithm of the minimal angle of resolution (logMAR) for the statistical analysis. The IOP was measured using a noncontact tonometer (Canon) and was calculated as the average value of 3 measurements.

### 2.4. Surgical Procedures

All participants were fully instructed on the use of the NRS assessments once they were enrolled. On the operation day, standard preoperative preparations were completed including the intramuscular injection of hemocoagulase 30 minutes before surgery. Patients in the K group received a 60 mg of intramuscular ketorolac 30 minutes before surgery.

Upon arrival in the operating room, routine monitoring was implemented, including electrocardiography, heart rate, noninvasive blood pressure, and pulse oximetry. A retrobulbar block was administered with a 3.5 mL injection of lidocaine and bupivacaine (2% lidocaine/0.75% bupivacaine; 50 : 50) into the conical retrobulbar space. We chose this dose for the consideration that the orbital size of Chinese population was generally smaller than the white population, and an overdose of anesthetics into the retrobulbar space may limit the ocular movement, as well as having a risk of toxic reactions [12].

The scleral buckling surgery followed a standard procedure [2, 4]. Briefly, after a 360° peritomy of the conjunctiva and the Tenon's capsule at the limbus, the following procedures were conducted: localization of the break(s), transscleral cryopexy under indirect ophthalmoscopy, drainage of the subretinal fluid (if necessary), and placement of a segmental silicone explant and an encircling band. Any intraoperative complications were recorded. The operations were performed by three professional surgeons (L.L., L.J.Q., and L.T.) with comparable surgical experience. All surgeries were completed within 1 hour.

### 2.5. Pain Score Assessment

For this trial, we adopted the 11-point (0–10) NRS for pain assessment, with a classification of the pain levels as follows [3, 12]:

- Level 0 = no pain
- Levels 1–3 = mild pain
- Levels 4–6 = moderate pain
- Levels 7–10 = severe pain.

The NRS assessment has been confirmed sensitive and reliable for eye pain evaluation in previous studies [3, 17]. Each participant was instructed to report their NRS scores for three times: before surgery, immediately after the operation, and 24 hours postoperatively.

At the baseline, none of the participants reported any pain. On the operation day, the participants were instructed to perceive their pain feelings throughout the whole surgical procedure and reported their intraoperative NRS scores immediately after completing the operation. Then, the participants were asked again to report their NRS scores 24 hours postoperatively, before they received any postoperative eye examinations.

### 2.6. Supplemental Analgesic Usage and Adverse Effects

During the postoperative period, a participant was given a 0.5 g of oral paracetamol when the postoperative NRS score is either 3 or above or to the demand of participants themselves. The number of patients who required rescue analgesics and the total consumption of paracetamol were recorded.

Any acute adverse events, for example, postoperative nausea and vomiting (PONV), were recorded. The severity of the PONV was evaluated as follows: 0 = none, 1 = nausea once, 2 = vomited once, and 3 = suffered from nausea twice or more or had ≥2 emetic episodes within 2 hours [18]. Metoclopramide was given when the participant's PONV score was 3. Any other adverse events were recorded during the operation and postoperatively.

### 2.7. Sample Size Calculation and Statistical Analysis

The sample size calculations were based on our pretrial outcome of intraoperative NRS. Given an equal randomization (1 : 1), the probability of type I error (*α*) is 5%, and the power (1–*β*) is 90%, to detect an NRS reduction of 2 or more between the K group and the P group. A total of 56 participants were required for the statistical significance.

Quantitative data were presented as the mean ± standard deviation (SD), confidence interval (CI), range, median, and interquartile range (IQR). Qualitative data were presented as the number of participants and the percentiles. Data of continuous variables were analyzed by the Student *t*-test or the Mann–Whitney *U* test, as appropriate. Categorical variables were analyzed by using the chi-square test. A value of *P* < 0.05 was set as the level of significance. All of the analyses were performed by using the SPSS 22.0 version (IBM, Armonk, NY).

## 3. Results

Beginning in July 2014, 73 eyes of 73 participants with RRD were recruited for this trial in our day care unit, and according to the inclusion and exclusion criteria, only 60 participants were then enrolled. The participants enrolled were randomly assigned (1 : 1) into the ketorolac group (K group) and the control group (C group). One participant in the C group failed to attend the surgery due to a fall accident before the operation. Therefore, there were actually 30 eyes of 30 participants in the K group and 29 eyes of 29 participants in the C group that took part in this clinical trial.

### 3.1. Demographic Characteristics and Ophthalmic Examinations

In general, the mean age of the participants was 33.5 ± 10.1 years old (95% Cl, 30.9–36.2; range, 18–54). There were 47 male participants (79.7%) and 12 female participants (20.3%), with a male to female ratio of nearly 4 : 1.

Preoperatively, all of the participants were phakic and the median BCVA (logMAR) was 0.92 (IQR, 0.52–1.40). The mean IOP was 12.0 ± 2.5 mmHg (95% Cl, 11.3–12.7; range, 7.0–19.0), and all of the participants' IOPs were within normal criteria.

Overall, the ages, genders, BCVAs, and IOPs were comparable between the K and C groups at the baseline (*P* > 0.05). The participants' demographic and ophthalmic characteristics in both groups are summarized in Table 1.

### 3.2. Comparison of Perioperative Pain

**Figure 2:**
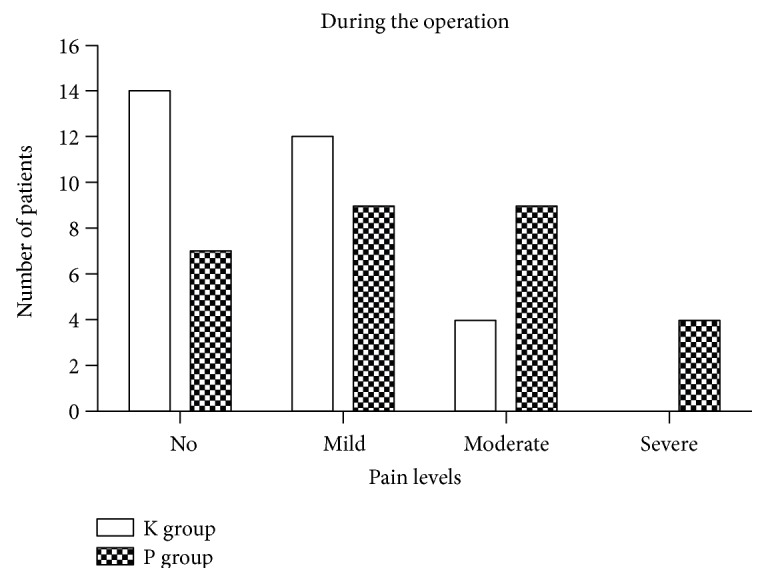
Number of participants with different levels of pain feelings in the K and C groups during the operation. Pain levels were defined by NRS scores: no = 0; mild = 1–3; moderate = 4–6; severe = 7–10.

**Figure 3:**
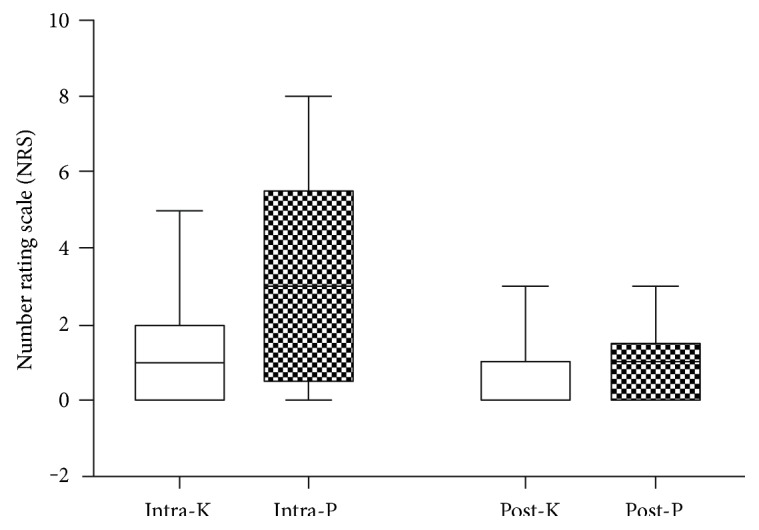
A comparison of NRS scores between the K and C groups both intraoperatively and postoperatively. Intra-K = intraoperative assessment in the ketorolac group; intra-C = intraoperative assessment in the control group; post-K = postoperative assessment in the ketorolac group; post-C = postoperative assessment in the control group.

There was no pain in any of the participants at the baseline. During the operation, 75.9% of the participants in the C group compared to 53.3% of those in the K group reported pain (*P* = 0.071) (Figure 2). The NRS score in the C group (median, 3; IQR, 0.5–5.5) was significantly higher than that in the K group (median, 1; IQR, 0.0–2.0; *P* = 0.003) (Figure 3). Thirteen of the participants in the C group (44.8%) compared to 4 participants in the K group (13.3%) reported moderate to severe pain (NRS ≥ 4) during the operation (*P* = 0.008). The highest NRS score in the C group was 8 in one patient lasting for less than 1 minute, while it was 5 in two patients in the K group lasting for less than 1 minute. Among those who reported pain, the most frequently reported NRS score in the C group was 4 (22.7%) and in the K group was 1 (37.5%). Postoperatively, 17 participants (58.6%) in the C group compared to 8 participants (26.7%) in the K group reported pain (*P* = 0.013), and no moderate to severe pain (NRS ≥ 4) was reported (Figure 3).

### 3.3. Postoperative Analgesic Consumption and Adverse Effect

Statistically significantly fewer participants required rescue analgesics postoperatively in the K group (10.0%) than in the C group (34.5%) (*P* = 0.023). None of the participants took more than once of rescue analgesics during the postoperative 24 hours. The total amount of paracetamol consumption in the K group was 1.5 g, and that in the C group was 5.0 g (Table 2).

The most complained postoperative adverse effects were the incidences of PONV (Table 2). The percentage of participants who reported PONV was significantly lower in the K group (13.3%) than in the C group (41.4%) (*P* = 0.015). Moreover, 100% of the participants who reported PONV scored 1 in the K group, but only 41.7% of those scored 1 in the C group. However, the PONV scores between the two groups were not statistically significant (*P* = 0.057). Besides, 3 participants in the C group had symptoms of dizziness, headache, or chest distress postoperatively, while only 1 participant in the K group reported dizziness. No gastrointestinal bleeding or other postoperative complications were observed.

During the operations, each group reported 1 eye with limited subretinal hemorrhages, and they were controlled by temporary IOP elevations. No other serious complications, such as ocular perforation or severe retrobulbar hemorrhage, were observed intraoperatively.

### 3.4. Surgical Outcome

The postoperative BCVA (logMAR) in the K group (median, 1.20; IQR, 0.88–1.43) and the C group (median, 1.00; IQR, 0.70–1.45) was slightly higher than those preoperatively (K group: median 1.07, IQR 0.48–1.50; C group: median 0.82; IQR 0.56–1.40) (Table 1). The postoperative BCVA changes (△BCVA) were not statistically different in the two groups (*P* = 0.412).

The postoperative IOP in the K group (median, 16.0 mmHg; IQR, 13.0–18.5 mmHg) was higher than before the surgery (median, 12.0 mmHg; IQR, 10.8–13.0 mmHg). The IOP changes (△IOP = postoperative IOP–preoperative IOP) in the C group were statistically higher than those in the K group (*P* = 0.038). During the postoperative ophthalmic examinations, 2 participants in the C group had ocular hypertension more than 30, and their IOPs were controlled by antiglaucoma drugs.

## 4. Discussion

According to our clinical trial, the combination of ketorolac with LA exhibited better pain control in day care scleral buckling surgery than LA alone. The administration of ketorolac lowered the incidence of supplementary analgesic consumption, was effective in reducing PONV, and may reduce postoperative elevation of IOP.

In our study, we observed mild to severe pain in 22 participants in the C group (75.9%) and 16 participants in the K group (53.3%). A percentage of 58.6% of participants in the C group and 26.7% in the K group reported pain postoperatively. This is consistent with other studies [3, 9, 19]; Marzak et al. [9] reported that 57.5% of their patients had postoperative pain and the greatest pain was during the first 4 hours after scleral buckling surgery. A survey of 100 RRD patients after scleral buckling surgery found that all of them reported eye pain on the first postoperative day [3], and 18% of the patients developed chronic eye pain. The investigators concluded that patients with more intense pain at the onset of the postoperative period tended to develop chronic eye pain.

Several factors contribute to perioperative eye pain. High-intensity noxious stimulation generated by the division of tissues, repeated ocular muscular traction [3, 9], manipulation and trauma to the globe and nearby tissues when planting segment silicone explants and an encircling band [10], cryopexy [10], and drainage of subretinal fluid can be the causes of primary phase injury. The noxious impulses from the surgery-induced tissue trauma reach the spinal cord, thus inducing central neural sensitization that amplifies the subsequent pain feelings [8]. The secondary phase of injury is mainly induced by inflammation [3, 8]. Manipulation of tissues and cryopexy could induce a breakdown of the blood-retinal barriers, thus releasing prostaglandins and other inflammatory mediators [20]. The inflammatory factors and released enzymes reduce the threshold for the activation of nociceptor neurons [8], thus causing feelings of pain. Moreover, an insufficient afferent blockade of local anesthesia may also be a source of pain [21, 22].

Ketorolac is an NSAID with a very strong analgesic effect. Ketorolac can inhibit cyclooxygenase- (COX-) 1 and COX-2 activities, which generate the inflammatory mediators such as prostaglandins [23]. As a result, ketorolac reduces the sensitivities of afferents and finally reduces pain feelings. Like other NSAIDs, ketorolac has side effects such as gastrointestinal hemorrhage, dyspepsia, headache, and so forth However, none of the participants in our trial was found any serious complications. We believe that one injection of ketorolac before surgery is safe to patients in scleral buckle surgery.

Our trial found a marked elevation of IOP in the C group than that in the K group postoperatively, and this was consistent with previous studies. For example, Soni et al. [1] had reported a high postoperative IOP in 21 of 280 patients who underwent scleral buckle surgery; Edmunds and Canning [24] observed that acetazolamide could significantly lower postoperative IOP after scleral buckle surgery. One of the main causes of postoperative IOP elevation was the intraocular inflammation due to massive tissue manipulation [3, 24]. Thus, the anti-inflammatory effect of ketorolac by inhibiting the cyclooxygenases (COXs) may partially lower the postoperative IOP elevation.

The results of our trial contribute to the popularization of day care scleral buckling surgery under LA. The wide spread of day care surgery from in-patient surgery has been taken place in recent decades [25, 26]. A day care surgery meets the patients' requirements by saving medical cost, shortening patients' waiting time, and simplifying the procedures without reducing the service qualities [25]. Besides, the surgical outcome is comparable to that of in-patient surgeries, [26, 27] with no additional risk of complications [28]. Moreover, the adoption of day care units greatly increases the utilization of beds, reduces medical resources waste, and enables surgeons to perform more surgeries at a certain time [25, 26]. The administration of LA contributes to the application of day care surgery by saving operation time, lowering systemic risks of anesthetic accidents, and reducing the potential danger during recovery in comparison to GA [7, 29, 30]. Besides, patients under LA have a quick recovery postoperatively and patient comfort is increased with the use of LA [29, 31]. Moreover, the administration of ketorolac adds to the feasibility and acceptability of day care scleral buckling surgery under LA.

There were some limitations in this study; for example, the participants enrolled were from a single center with a relatively small population. Secondly, the results of our trial cannot be applied to those patients who have contraindications to the drugs.

Our clinical trial does exhibit several strengths; for example, it was a well-designed, prospective, controlled study. Secondly, we adopted the reliable NRS to assess pain. Thirdly, we assess intraoperative eye pain other than the commonly studied postoperative pain for there was a high incidence of pain during the surgery. Finally, we gave our participants oral paracetamol for postoperative pain relief since it does not belong to the NSAIDs and it is easier to administer for an outpatient setting.

## 5. Conclusions

In conclusion, perioperative ocular pain is a common but often underestimated issue for scleral buckling surgery [3]. The combination of ketorolac with conventional LA is effective in providing better anesthesia, reducing pain, supplementary analgesics, and PONV. Moreover, ketorolac may lower postoperative elevation of IOP. The results of our study are encouraging for the practice of LA in outpatient scleral buckling surgery.

## Figures and Tables

**Table 1:** Demographic and ophthalmic characteristics of patients.

Variable	K group (*n* = 30)	P group (*n* = 29)	*P* value
Age (years)			
Mean ± SD	34.5 ± 10.2	32.6 ± 10.2	0.473^∗^
Gender			
Male, number (%)	23 (76.7)	24 (82.8)	0.797^∗∗^
Educational level, number (%)			0.544^∗∗^
0	15 (50)	14 (48.3)	—
1	6 (20)	9 (31.0)	—
2	9 (30)	6 (20.7)	—

Preoperative ophthalmic characteristics			
BCVA (logMAR)			
Mean ± SD	1.05 ± 0.65	1.11 ± 0.91	—
Median (IQR)	1.07 (0.48–1.50)	0.82 (0.56–1.40)	0.808^∗∗∗^
IOP (mmHg)			
Mean ± SD	12.1 ± 2.7	11.9 ± 2.4	0.819^∗^
Median (IQR)	12.0 (10.2–14.0)	12.0 (10.8–13.0)	—

Postoperative ophthalmic characteristics			
BCVA (logMAR)			
Mean ± SD	1.22 ± 0.52	1.26 ± 0.81	—
Median (IQR)	1.20 (0.88–1.43)	1.00 (0.70–1.45)	—
△BCVA (logMAR), median (IQR)	0.00 (−0.11–0.33)	0.18 (−0.06–0.54)	0.412^∗∗∗^
IOP (mmHg)			
Mean ± SD	13.9 ± 4.2	17.1 ± 6.3	—
Median (IQR)	13.0 (10.0–16.3)	16.0 (13.0–18.5)	—
△IOP (mmHg), median (IQR)	1.9 (−1.3–4.0)	3.0 (1.5–6.4)	0.038^∗∗∗^

K group: ketorolac group; P group: placebo group; BCVA: best-corrected visual acuity; logMAR: logarithm of the minimum angle of resolution; IOP: intraocular pressure; IQR: interquartile range; SD: standard deviation; △BCVA = postoperative BCVA–preoperative BCVA; △IOP = postoperative IOP–preoperative IOP; ^∗^*t*-test; ^∗∗^Chi-square test; ^∗∗∗^Mann–Whitney *U* test.

**Table 2:** Comparison of postoperative analgesic consumption and adverse effect between the ketorolac administration group and placebo group.

Variable	K group (*n* = 30)	P group (*n* = 29)	*P* value
Postoperative analgesic usage			0.023^∗^
None, number (%)	27 (90)	19 (65.5)	—
Analgesic use, number (%)	3 (10)	10 (34.5)	—
Supplemental analgesic consumption			
Paracetamol (g)	1.5	5.0	—
Adverse effect			
PONV, number (%)	4 (13.3)	12 (41.4)	0.015^∗^
PONV score, number			0.057^∗^
1	4	5	—
2	0	5	—
3	0	2	—
Total metoclopramide consumption (mg)	0	20	—

K group: preoperative ketorolac group; P group: preoperative placebo group; PONV: postoperative nausea and vomiting; ^∗^chi-square tests.
